# Unraveling heterogeneity within ACPA-negative rheumatoid arthritis: the subgroup of patients with a strong clinical and serological response to initiation of DMARD treatment favor disease resolution

**DOI:** 10.1186/s13075-021-02671-z

**Published:** 2022-01-03

**Authors:** M. Verstappen, H. W. van Steenbergen, P. H. P. de Jong, A. H. M. van der Helm-van Mil

**Affiliations:** 1 Department of Rheumatology, Leiden University Medical Centre, Leiden, the Netherlands; 2 Department of Rheumatology, Erasmus Medical Centre, Rotterdam, the Netherlands

## Abstract

**Background:**

Rheumatoid arthritis (RA) is a heterogeneous disease, as evidenced by the differences in long-term outcomes. This applies especially to anti-citrullinated protein antibodies (ACPA)-negative RA, where a proportion achieves sustained DMARD-free remission (SDFR; sustained absence of synovitis after DMARD cessation). Differentiation of RA patients who will achieve SDFR can guide personalized treatment/tapering strategies. Although this subgroup remains scarcely discerned, previous research demonstrated that these RA patients are characterized by an early clinical response (DAS remission after 4 months) after DMARD start. We studied whether, in addition to this clinical response, a specific biomarker response can further distinguish the subgroup of RA patients most likely to achieve SDFR.

**Methods:**

In 266 RA patients, levels of 12 biomarkers (SAA/CRP/MMP-1/MMP-3/resistin/leptin/IL-6/TNF-R1/YKL-40/EGF/VEGF/VCAM-1), in the first 2 years after diagnosis, were studied in relation to SDFR, stratified for ACPA status. Subsequently, biomarkers associated with SDFR development were combined with early DAS remission to study its additional value in defining subgroups. Since most biomarker levels are not routinely measured in clinical practice, we explored how this subgroup can be clinically recognized.

**Results:**

ACPA-negative RA patients achieving SDFR were characterized by high baseline levels and stronger decline in MMP-1/MMP-3/SAA/CRP after DMARD-start, respectively 1.30×/1.44×/2.12×/2.24× stronger. This effect was absent in ACPA-positive RA. In ACPA-negative RA, a strong biomarker decline is associated with early DAS remission. The combination of both declines (clinical, biomarker) was present in a subgroup of ACPA-negative RA patients achieving SDFR. This subgroup can be clinically recognized by the combination of high baseline CRP levels (≥ 3 times ULN), and early DAS remission (DAS_4 months_ < 1.6). This latter was replicated in independent ACPA-negative RA patients.

**Conclusions:**

ACPA-negative RA patients with early DAS remission and a strong biomarker response (or baseline CRP levels ≥ 3× ULN) are most likely to achieve SDFR later on. This could guide personalized decisions on DMARD tapering/cessation in ACPA-negative RA.

**Supplementary Information:**

The online version contains supplementary material available at 10.1186/s13075-021-02671-z.

## Introduction

Rheumatoid arthritis (RA) is an auto-immune syndrome which, from a pathophysiological perspective, presumably consists of different disease entities. In this, it has been suggested that ACPA-positive and ACPA-negative RA might be considered as separate subgroups of RA [1, 2]. Yet, heterogeneity within these subgroups remains, especially among ACPA-negative RA patients. Although ACPA-negative RA is considered a milder disease than ACPA-positive RA, long-term outcomes diverge more widely between ACPA-negative RA patients [2]. Sustained DMARD-free remission (SDFR; sustained absence of synovitis after DMARD discontinuation) is prevalent within ACPA-negative RA (~ 40%), but conversely, other ACPA-negative RA patients have persisting disease, generally requiring life-long disease-modifying antirheumatic drugs (DMARDs) [3]. The course of this group of ACPA-negative RA patients resembles ACPA-positive RA, where SDFR can only be achieved by ~ 5–10%, and persistent or progressive disease is common [4, 5]

Identification of ACPA-negative RA patients who can achieve SDFR would be clinically relevant, for instance, to achieve a more tailor-made tapering approach in RA. However, the identification of a subgroup of ACPA-negative RA patients who are most likely to achieve SDFR has proven to be extremely difficult [6]. Clinical and imaging characteristics at the time of diagnosis appeared to be mostly similar in ACPA-negative RA patients that achieve SDFR and those who do not [7, 8].

Recently two encouraging findings were done. First, a study on serological biomarkers demonstrated that the subgroup of ACPA-negative RA patients achieving SDFR is characterized by higher levels of inflammatory markers (SAA, CRP) and matrix metalloproteinase-3 (MMP-3) at diagnosis [9]. Second, in ACPA-negative RA, a stronger DAS response in the first 4 months after DMARD initiation, resulting in early DAS remission (DAS_4 months_ < 1.6), was associated with a higher change at SDFR development. On the contrary, ACPA-negative RA patients with DAS_4 months_ ≥ 3.6 rarely achieved SDFR (< 5%) [5]. Although both findings insufficiently characterize the ACPA-negative RA patients who will achieve SDFR, these findings prompted the hypothesis that ACPA-negative RA patients achieving SDFR might also have a stronger biomarker response after DMARD treatment. And if we combine both, this may further distinguish a subgroup among ACPA-negative RA patients with a high likelihood of achieving SDFR. Ultimately, this understanding might help to unravel the heterogeneity within ACPA-negative RA.

**Fig. 1:**
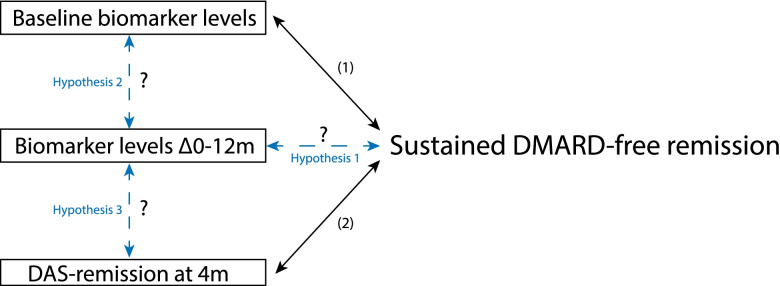
Schematic depiction of the three hypotheses that were explored. Previous research showed that high biomarker levels of MMP-3, SAA, and CRP were associated with the development of sustained DMARD-free remission (SDFR) in ACPA-negative RA (1) [9]. Other research showed that ACPA-negative RA patients achieving early DAS remission, i.e., DAS_4 months_ < 1.6 after DMARD initiation, were more likely to achieve SDFR (2) [5]. Combining these previous findings, we studied whether the decline in biomarker levels in the first year after diagnosis was also associated with SDFR (hypothesis 1). Moreover, we explored whether baseline biomarker levels (hypothesis 2) and DAS remission (hypothesis 3) were associated with the decline in biomarker levels in the first year of therapy. It should be noted that this figure is used to illustrate the defined hypothesis and does not suggest any causality

In this study, we combined the previous findings on baseline biomarker levels and early DAS remission with novel data on biomarker levels over time, with the ultimate aim to identify a subgroup of RA patients most likely to achieve SDFR. Yet, we addressed the following complementary hypotheses: (1) the biomarker response in MMP-3, SAA, and CRP after DMARD initiation is stronger in ACPA-negative RA patients achieving SDFR; (2) a strong biomarker response in MMP-3, SAA, and CRP occurs in the same RA patients with high baseline levels of these biomarkers; and (3) a combination of a strong biomarker response and early DAS remission occurs in ACPA-negative RA patients who achieve SDFR (Fig. 1).

## Patients and methods

### Study population

Patients were obtained from the Leiden Early Arthritis Clinic (EAC), which has been previously described [10]. In short, the Leiden EAC is an inception cohort, including all patients presenting with recent-onset arthritis with a symptom duration ≤ 2 years. Research visits took place at baseline, after 4 months and 8 months, and annually thereafter. During these visits, joint counts were performed, disease activity scores calculated [11], laboratory measurements performed, and questionnaires filled out. Visits to the treating rheumatologists were more frequent, as often as it was found necessary.

Biomarker measurements were performed in consecutive RA patients included in the Leiden EAC between 2010 and 2015, representing the period in which early methotrexate (MTX) and treat-to-target strategies were common. RA was stringently defined by a clinical diagnosis of RA by an experienced rheumatologist plus fulfillment of the 1987 and/or 2010 criteria [12, 13]. Patients diagnosed with conditions other than RA (e.g., reactive arthritis/psoriatic arthritis/inflammatory osteoarthritis), or who had a high suspicion on these diagnoses, were excluded. Biomarker levels were measured in 312 RA patients. RA patients who did not use DMARDs during follow-up (*n* = 13), who had a delayed DMARD start (> 4 months after diagnosis) (*n* = 16), or who concomitantly participated in a clinical trial (*n* = 17), and therefore not routinely, were excluded. Consequently, 266 RA patients were selected as the primary study population, of whom 135 were ACPA-positive and 131 were ACPA-negative RA patients (Fig. S1). The baseline characteristics of the study population and RA patients excluded from the study population did not remarkably differ (S2).

Second, for the replication of findings, ACPA-negative RA patients, consecutively included in the Leiden EAC between 2007 and 2010, were studied (Fig. S1). The same criteria for the classification of RA and patient selection were applied as described above.

### Treatment

Treatment strategies in the Leiden EAC are described elsewhere [2]. In brief, all included RA patients were promptly treated with conventional synthetic DMARDs (csDMARDs) after diagnosis; methotrexate (MTX) was the first choice. Subsequently, DAS-steered treatment adjustments were made. When the initial treatment failed, another csDMARD was initiated or added. A biological DMARD (bDMARD) was allowed when RA patients failed ≥ 2 csDMARDs. When low disease activity (DAS44 < 2.4) was sustained, and clinical synovitis was absent, treatment could be tapered and eventually discontinued. Guidelines were to taper DMARDs in case of DAS44 < 2.4 in subsequent visits to the rheumatologist; however, decisions on DMARD cessation were taken in shared decision-making between rheumatologists and patients.

### Outcome

Sustained DMARD-free remission (SDFR) was defined as the absence of clinical synovitis (swollen joints at physical examination) after cessation of DMARD treatment (including systemic/intra-articular corticosteroids) that persisted for the entire follow-up thereafter, and this follow-up should be ≥ 1 year. RA patients experiencing a late flare (defined as reoccurrence of clinical synovitis) after SDFR development were also included in the non-SDFR group. These stringent definitions were chosen to ensure the sustainability of DMARD-free remission. Medical files were studied on the occurrence of SDFR until August 2020.

### Biomarker measurements

Serum samples were collected at disease presentation (before DMARD initiation, including corticosteroids) and after 12 and 24 months (stored at − 80 °C). Biomarker levels were determined using three separate multiplex, sandwich immunoassays (as previously described) [14]. Measurements were performed blinded to clinical data and outcome.

A previous biomarker study found that baseline levels of three out of twelve studied biomarkers were associated with SDFR development: MMP-3, SAA, and CRP [9]. We hypothesized that the levels of these three biomarkers might subsequently change differently over time in RA patients achieving SDFR compared to those who do not (hypothesis 1, Fig. 1). Therefore, these biomarkers were the main subject of this study. The other 9 biomarkers from this previous study, matrix metalloproteinase-1 (MMP-1), tumor necrosis factor receptor superfamily member-1A (TNF-R1), interleukin-6 (IL-6), leptin, resistin, human cartilage glycoprotein-39 (YKL-40), epidermal growth factor (EGF), vascular endothelial growth factor-A (VEGF-A), and vascular cell adhesion molecule-1 (VCAM-1), were also evaluated and considered as “negative controls” to preclude that changes reflected regression-to-the-mean. Thus, for these biomarkers, we did not presume to find changes related to achieving SDFR.

### Statistical analysis

The statistical methods are extensively described in supplementary S3. In short, to test our first hypothesis (Fig. 1), the course of the individual biomarkers was compared between the SDFR group and non-SDFR group using linear mixed models (LMM), stratified for ACPA status. Since biomarker levels were measured at three specific time points (0/12/24 months), differences in biomarker course between the SDFR groups were compared for two time periods: 0–12 months/12–24 months. Biomarker levels were log-transformed because of non-normal distribution. Because logarithmic results are measured on a multiplicative scale, differences in biomarker course between the SDFR group and non-SDFR group, in the two specific time periods, were expressed as ratios.

Of the included 266 RA patients, follow-up biomarker measurements were not available in 42 RA patients. Missing biomarker measurements were not imputed or excluded since LMM analysis can handle missing data, assuming missingness is at random. Baseline characteristics of these 42 RA patients did not remarkably differ from patients who did have follow-up biomarker data (S4).

As it was previously observed that biomarker levels at diagnosis were related to SDFR-development [9], correlations between baseline biomarker levels and decline in levels in the first 12 months were plotted (testing hypothesis 2, Fig. 1) and expressed using Spearman’s rho. Additionally, considering the previous findings on the relation between early DAS remission (DAS_4 months_ < 1.6) and SDFR [5], median biomarker change within the first 12 months was compared between RA patients with and without early DAS remission using the Mann-Whitney *U* test (testing hypothesis 3, Fig. 1).

Several sub-analyses were carried out. First, RA patients achieving SDFR within 3 years of follow-up were excluded from the analyses. From all RA patients achieving SDFR, these were the RA patients who reached SDFR after the shortest disease duration. If overtreatment (treatment of patients who might otherwise have had spontaneous resolution) would be an issue, these excluded patients would presumably be part of this group. Second, to explore whether the effects differed per initial DMARD, analyses were repeated in the subgroup of RA patients initially treated with MTX, as this was the most frequently prescribed initial DMARD. Finally, ACPA-negative rheumatoid factor (RF)-positive RA patients were excluded to restrict analyses to autoantibody-negative RA patients.

STATA (V16) was used. *p*-values < 0.05 were considered statistically significant.

## Results

### Primary study population

A total of 266 RA patients were studied: 131 ACPA-negative and 135 ACPA-positive RA patients. The median follow-up duration was 7.2 years (IQR 6.5–8.6). During this follow-up, 72 RA patients achieved SDFR after a median follow-up of 3.6 years (IQR 2.8–4.9). Notably, RA patients achieving SDFR were subsequently followed for a median of 3.8 years (IQR 2.3–5.0) after SDFR development. SDFR was achieved in 48.1% of ACPA-negative RA patients (*n* = 63) and in 6.7% of ACPA-positive RA patients (*n* = 9). At baseline, ACPA-negative RA patients achieving SDFR were slightly older and less often RF-positive (Table 1). The baseline characteristics of the ACPA-positive study population are described in supplementary S5.

**Table 1:** Baseline characteristics ACPA-negative RA study population

**A**	**ACPA-negative RA (*n*** **= 131)**	**Non-SDFR group (*n*** **= 68)**	**SDFR group (*n*** **= 63)**
Age (years), mean (SD)	61 (15)	57 (16)	65 (11)
Females, *n* (%)	66	66	65
RF positivity, *n* (%)	34	47	19
Symptom duration at diagnosis (≤ 12 weeks), *n* (%)	45	41	50
DAS at baseline, med (IQR)	3.5 (2.7–4.4)	3.4 (2.7–4.2)	3.6 (2.7–4.6)
SJC at baseline (0–44), med (IQR)	8 (3–12)	8 (3–11)	8 (3–13)
TJC at baseline (0–53), med (IQR)	9 (4–19)	10 (4–19)	9 (4–17)
ESR (mm/h), med (IQR)	28 (11–39)	21 (13–37)	29 (11–43)
VAS (0–100 mm), med (IQR)	50 (30–70)	50 (30–60)	60 (30–80)
HAQ, med (IQR)	1.1 (0.6–1.5)	1.1 (0.6–1.5)	1.1 (0.6–1.6)
**B**	**ACPA-negative replication population (*n*** **= 95)**	**Non-SDFR group (*n*** **= 50)**	**SDFR group (*n*** **= 45)**
Age (years), mean (*SD*)	61 (17)	60 (17)	63 (17)
Females, *n* (%)	62	66	58
RF positivity, *n* (%)	12^*^	14	10
Symptom duration at diagnosis (≤ 12 weeks), *n* (%)	44	53	34
DAS at baseline, med (IQR)	3.6 (3.0–4.2)	3.6 (3.0–4.2)	3.7 (3.1–4.4)
SJC at baseline, (0–44), med (IQR)	8 (3–11)	6 (3–10)	8 (4–14)
TJC at baseline, (0–53), med (IQR)	13 (8–18)	12 (7–18)	13 (10–18)
ESR (mm/h), med (IQR)	22 (9–46)	19 (9–41)	22 (11–49)
VAS (0–100 mm), med (IQR)	32 (14–54)^*^	29 (10–52)	35 (16–18)
HAQ, med (IQR)	1.0 (0.6–1.8)	1.1 (0.8–1.8)	0.9 (0.6–1.6)

Baseline characteristics of the ACPA-negative study population (A) and of the ACPA-negative replication population (B), stratified for SDFR development. Baseline characteristics of the ACPA-negative study population and the replication population were comparable

*DAS*, disease activity score based on swollen joint count (44 joints), tender joint count (53-joints), ESR, and pain; *SJC*, swollen joint count; *TJC*, tender joint count; *ESR*, estimated sedimentation rate; *VAS*, visual analog scale; *RF*, rheumatoid factor

^*^Statistically significant difference (*p*-value < 0.05) between the ACPA-negative study population and the ACPA-negative validation population

### Biomarker levels over time in relation to SDFR

**Fig. 2:**
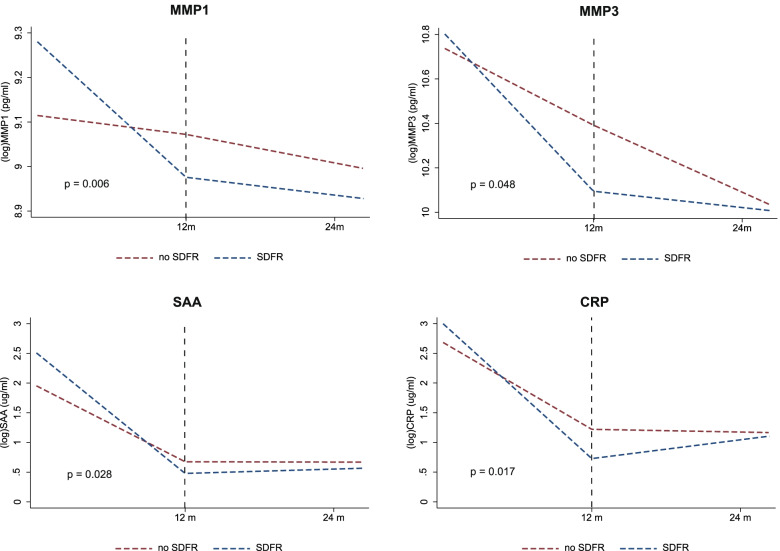
Modeled course of MMP-1, MMP-3, SAA, and CRP levels in the first 24 months after DMARD initiation, in ACPA-negative RA patients achieving SDFR and those who did not achieve SDFR. Biomarker levels were measured at baseline, 12 months, and 24-months and modeled to visualize the differences between the SDFR and non-SDFR groups between baseline and 12 months and between 12 and 24 months. Before modeling, biomarker levels were log-transformed. In the SDFR group, MMP-1, MMP-3, SAA, and CRP showed a statistically significant stronger decline in the first 12 months after DMARD start compared to the non-SDFR group. Vertical dotted lines indicate the transition from one time period (0–12 months) to the following (12–24 months) which were separately estimated. CRP, C-reactive protein; MMP, matrix metalloproteinase; SAA, serum A amyloid; SDFR, sustained DMARD-free remission

ACPA-negative RA patients achieving SDFR had a stronger decline in levels of MMP-3, SAA, and CRP in the first 12 months after treatment initiation, compared to ACPA-negative RA patients who did not achieve SDFR (Fig. 2, Table 2), supporting hypothesis 1 (Fig. 1). Also, levels of MMP-1 demonstrated a stronger decline in the first 12 months in ACPA-negative RA patients achieving SDFR, compared to those who did not. The decline of the other biomarkers (IL-6/TNF-R1/resistin/leptin/YKL-40/EGF/VEGF/VCAM-1) did not differ over time between both patient groups (S6). The decline in MMP-1 and MMP-3 in the SDFR group was respectively 1.30× (95%CI 1.08–1.57; *p* = 0.006) and 1.44× (95%CI 1.00–2.06; *p* = 0.048) stronger than in the non-SDFR group (Table 2). In SAA and CRP, this decline was respectively 2.12× (95%CI 1.08–4.14; *p* = 0.028) and 2.24× (95%CI 1.16–4.35; *p* = 0.017) stronger in patients achieving SDFR (S6).

**Table 2:** Decline in biomarker levels over time in ACPA-negative RA patients achieving SDFR compared to those who did not

	No SDFR, ***β*** (95%CI)	SDFR, ***β*** (95%CI)	***p***-value	Decline in SDFR vs. non-SDFR, ratio (95%CI)
**MMP-3 (log-pg/ml)**				
*Baseline*	10.74 (10.54, 10.94)	10.80 (10.31, 11.29)	0.660	
*Change 0–12 months*	− 0.35 (− 0.60, − 0.09)	− 0.71 (− 0.96, − 0.45)	**0.048**	**1.44× (1.00**–**2.06)**
*Change 12–24 months*	− 0.28 (− 0.55, + 0.00)	− 0.07 (− 0.35, + 0.22)	0.305	0.81× (0.55–1.21)
**MMP-1 (log-pg/ml)**				
*Baseline*	9.12 (8.95, 9.28)	9.28 (8.88, 9.68)	0.174	
*Change 0–12 months*	− 0.04 (− 0.17, + 0.09)	− 0.30 (− 0.44, − 0.17)	**0.006**	**1.30× (1.08**–**1.57)**
*Change 12–24 months*	− 0.06 (− 0.20, + 0.09)	− 0.04 (− 0.19, + 0.11)	0.836	0.98× (0.80–1.20)
**SAA (log-μg/ml)**				
*Baseline*	1.95 (1.62, 2.29)	2.51 (1.70, 3.17)	0.023	
*Change 0–12 months*	− 1.28 (− 1.75, − 0.81)	− 2.03 (− 2.50, − 1.55)	**0.028**	**2.12× (1.08–4.14)**
*Change 12–24 months*	− 0.00 (− 0.53, + 0.52)	+ 0.07 (− 0.47, + 0.60)	0.851	0.93× (0.44–0.93)
**CRP (log-μg/ml)**				
*Baseline*	2.68 (2.34, 3.03)	3.00 (2.15, 3.84)	0.216	
*Change 0–12 months*	− 1.46 (− 1.93, − 1.00)	− 2.27 (− 2.74, − 1.80)	**0.017**	**2.24× (1.16–4.35)**
*Change 12–24 months*	− 0.04 (− 0.56, + 0.47)	+ 0.32 (− 0.24, + 0.82)	0.379	0.72× (0.34–1.50)

Estimated marginal means for the decline in biomarker levels between baseline and 12 months and between 12 and 24 in relation to SDFR development in ACPA-negative RA patients. Estimates are presented on a logarithimic scale, except for the most right column in which the relative change in biomarker levels in the SDFR is visualized, compared to the non-SDFR group. Ratio's are calculated as: 1 divided by the exponentiated difference in logarithmic decline

*CRP*, C-reactive protein; *MMP*, matrix metalloproteinase; *SAA*, serum amyloid A; *SDFR*, sustained DMARD-free remission

In ACPA-positive RA patients, no significant differences were seen in the course of MMP-1, MMP-3, SAA, and CRP levels between patients achieving SDFR and those who did not, although SDFR occurred infrequently (S7).

### Biomarker response in relation to biomarker levels at diagnosis

**Fig. 3:**
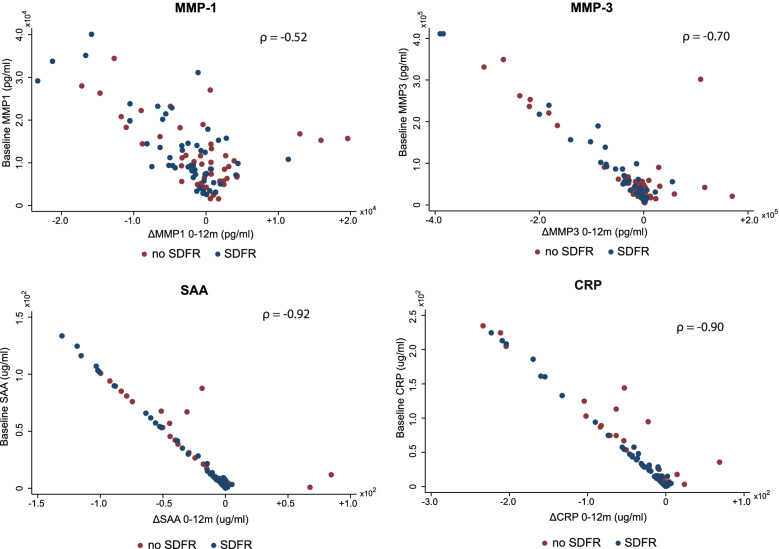
In APCA-negative RA, high baseline levels of MMP-3, SAA, and CRP are related to a strong decline in these levels after DMARD start. Correlation between baseline levels and change in the levels between baseline and 12 months, separately for the SDFR and non-SDFR group. *ρ* reflects Spearman’s rho. CRP, C-reactive protein; MMP, matrix metalloproteinase; SAA, serum A amyloid; SDFR, sustained DMARD-free remission

In ACPA-negative RA patients, high baseline levels of MMP-3, SAA, and CRP were highly correlated with a subsequent strong decline in the first year (correlation coefficients: 0.70/0.92/0.90) (Fig. 3). In MMP-1, this was less prominent (0.52). Thus, the subgroup of ACPA-negative RA patients which achieves SDFR is characterized by higher levels of MMP-3, SAA, and CRP at the time of diagnosis and a subsequent stronger decline in these levels after DMARD initiation, supporting hypothesis 2 (Fig. 1). In the other eight biomarkers, baseline levels and subsequent decline within the first year were less correlated (correlation coefficients ranging 0.03–0.65; Fig. S8), except for IL-6 (correlation coefficient 0.91).

### Biomarker response combined with early DAS remission associated with SDFR in ACPA-negative RA

**Fig. 4:**
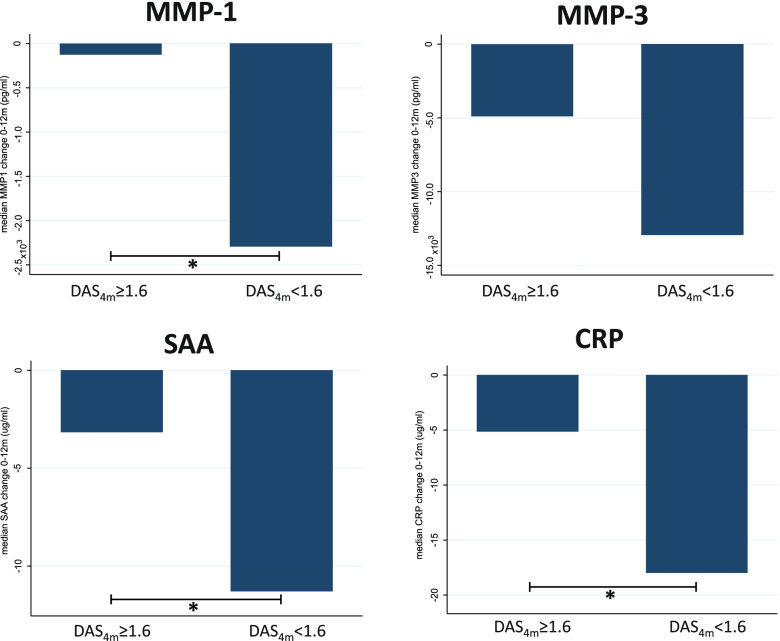
Biomarker response after DMARD start is stronger in ACPA-negative RA patients with early DAS remission. ACPA-negative RA patients with a DAS_4 months_ < 1.6 demonstrated a significantly stronger median decline in MMP-1, SAA, and CRP in the first year after DMARD initiation. *p < 0.05. CRP, C-reactive protein; MMP, matrix metalloproteinase; SAA, serum A amyloid; SDFR, sustained DMARD-free remission

**Fig. 5:**
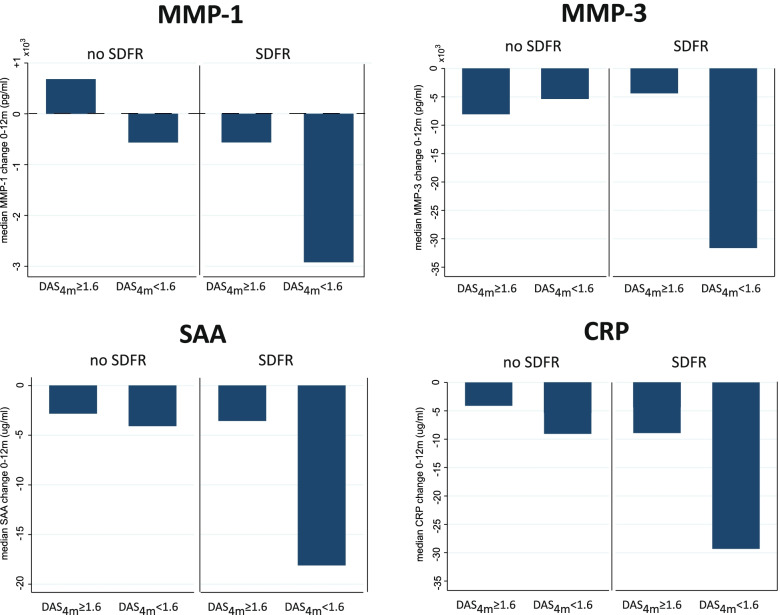
The combination of early DAS remission and a strong biomarker response after DMARD start was confined to a subgroup of ACPA-negative patients that achieved SDFR. ACPA-negative RA patients achieving SDFR are characterized by a strong biomarker response concomitant with early DAS remission (DAS_4 months_ < 1.6). This comprised 23% of all ACPA-negative RA patients. Only RA patients with both clinical and biomarker follow-up information could be included. The distribution of the numbers of patients per group is as follows: of the 50 ACPA-negative RA patients not achieving SDFR, 16 (32%) achieved DAS_4 months_ < 1.6, whereas 22 (48%) of the 46 ACPA-negative RA patients achieving SDFR achieved DAS_4 months_ < 1.6. CRP, C-reactive protein; MMP, matrix metalloproteinase; SAA, serum A amyloid; SDFR, sustained DMARD-free remission

When exploring the relation between a strong biomarker response and early DAS remission (hypothesis 3), we found that early DAS remission tended to be associated with a stronger median decline in MMP-1, MMP-3, SAA, and CRP in ACPA-negative RA patients achieving SDFR (Figs. 4 and 5, S9). In ACPA-negative RA patients not achieving SDFR, early DAS remission was not associated with a stronger biomarker decline in the first year. Moreover, the combination of early DAS remission and a strong biomarker response appeared to define a subgroup of ACPA-negative RA that achieves SDFR. In ACPA-positive RA, this combination did not relate to SDFR development (S9).

### Translation to clinical practice

**Fig. 6:**
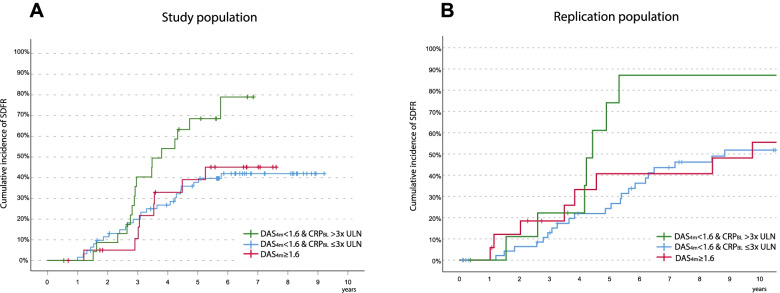
Cumulative incidence of SDFR was highest among ACPA-negative RA patients that presented with baseline CRP levels 3 times the ULN in both the study population as the replication population. Kaplan-Meier curve showing that SDFR is highly prevalent in ACPA-negative RA patients with relatively high baseline CRP levels (≥ 3× ULN) and early DAS remission (DAS_4 months_ < 1.6) in both the study population (**A**, *p* = 0.02) and the replication population (**B**, 0.03). In ACPA-negative RA patients with early DAS remission but baseline CRP levels < 3× ULN, the frequency of SDFR is comparable with ACPA-negative RA patients with DAS_4 months_ ≥ 1.6. BL, baseline; CRP, C-reactive protein; DAS, disease activity score; SDFR, sustained DMARD-free remission

Although a combination of a strong biomarker response and early DAS remission seems to indicate a subgroup of ACPA-negative RA that is likely to achieve SDFR, MMP3/MMP-1/SAA are not routinely measured in clinical practice and are therefore not feasible to clinically distinguish this subgroup. CRP, in contrast, is routinely measured. The identified subgroup had relatively high baseline CRP levels (Table 3). Since we demonstrated that CRP response was highly associated with baseline CRP levels (Fig. 3), we studied whether a combination of high baseline CRP (defined as ≥ 3× ULN, i.e., ≥ 15 mg/l, to exclude slight elevations above the local cutoff) and early DAS remission could clinically discriminate the subgroup with a high likelihood of achieving SDFR. We found that ~ 80% of ACPA-negative RA patients with high CRP levels at diagnosis and early DAS remission achieved SDFR, whereas this was only ~ 45% in the ACPA-negative RA patients with early DAS remission but without CRP ≥ 3 times ULN and in ACPA-negative RA patients without early DAS remission (*p* = 0.02, Fig. 6A). ACPA-negative RA patients with early DAS remission, but without CRP levels ≥ 3× ULN at diagnosis, achieved SDFR as frequent as ACPA-negative RA patients without early DAS remission, which further supports the notion that the combination of both high CRP at diagnosis and early DAS remission is most discriminative.

**Table 3:** Baseline characteristics of the identified subgroup of ACPA-negative RA compared to the remaining ACPA-negative RA patients

	SDFR and DAS_**4 months**_ < 1.6	SDFR and DAS_**4 months**_ ≥ 1.6	No SDFR and DAS_**4 months**_ > 1.6	No SDFR and DAS_**4 months**_ ≥ 1.6
**Age** (years), mean (*SD*)	**64 (12)**	64 (11)	60 (14)	59 (14)
**Gender** (female), %	**55**	21	33	50
**Sympt. duration (≤ 12 weeks)**, %	**64**	38	33	56
**RF-positive**, %	**27**	21	45	50
**DAS44 at baseline**, median (IQR)	**3.2 (2.5–4.1)**	3.6 (3.1–4.5)	3.4 (3.0–4.1)	2.8 (2.4–3.8)
**HAQ-DI at baseline**, median (IQR)	**1.0 (0.6–1.6)**	1.3 (0.8–1.6)	1.3 (0.9–1.5)	0.7 (0.3–1.2)
**SJC (0–44) at baseline**, median (IQR)	**8 (3–13)**	7 (3–11)	8 (2–11)	5 (2–11)
**TJC (0–53) at baseline**, median (IQR)	**5 (3–10)**	9 (6–19)	11 (6–19)	7 (3–11)
**ESR at baseline**, median (IQR)	**29 (22–38)**	34 (6–60)	22 (11–36)	18 (10–41)
**VAS at baseline (0–100)**, median (IQR)	**60 (30–80)**	60 (40–80)	40 (30–60)	45 (20–55)
**CRP at baseline (μg/ml)**, median (IQR)	**18.0 (8.7–26.3)**	11.0 (3.0–22.7)	7.0 (3.0–28.5)	9.3 (5.0–46.9)

Baseline characteristics of the identified subgroup (first column, bold font), compared to the remaining ACPA-negative RA population. Since numbers were low (*n* = 22, 24, 34, 16) between the different groups, statistical differences between these groups were not tested

Between 2007 and 2010, 375 RA patients were included in the Leiden EAC of which 95 consecutive ACPA-negative RA patients could be selected as replication population for this study (Table 1). Also within the replication population, SDFR incidence was highest among ACPA-negative RA patients with high baseline CRP levels (> 3× ULN) and early DAS remission, respectively 87% compared to ~ 52% in the other two groups (*p* = 0.03, Fig. 6B).

### Sub-analyses

Sub-analyses excluding ACPA-negative RA patients achieving SDFR within 3 years of follow-up yielded similar effect sizes although statistical significance was not achieved, presumably due to low numbers (S10). Analyses of solely the ACPA-negative RA patients initially treated with MTX yielded similar results as the primary analyses, as did analyses excluding RF-positive ACPA-negative patients (S11/S12).

## Discussion

We identified that a subgroup of ACPA-negative RA patients with a strong biomarker response after DMARD initiation and early DAS remission, has the highest capability of achieving the most favorable outcome in RA: disease resolution. This subgroup can be clinically recognized by the combination of high CRP levels at diagnosis and early DAS remission (DAS_4 months_ < 1.6). The fact that this finding was confirmed in an independent set of ACPA-negative RA patients provides some evidence for the robustness of the results. These findings may be feasible for use in clinical practice and can contribute to personalized treatment and tapering strategies in ACPA-negative RA.

Although the heterogeneity among RA is evident, so far, recommendations for DMARD initiation, as well as DMARD tapering, are similar for all RA patients [15]. Our findings suggest that 4 months after diagnosis and DMARD initiation, within a subgroup of ACPA-negative RA patients, the chances at successful tapering and discontinuation of DMARD treatment later on can be approximated. The optimal moment for DMARD tapering remains to be determined in future research. In this study, where DMARD tapering was non-protocolized, patients stopped DMARDs after a median treatment period of 2.6 years (IQR 1.8–3.9). In line with our definition, SDFR was achieved after a median of 3.6 years (IQR 2.8–4.9). Importantly, in the subsequent follow-up thereafter (median 3.8 years (IQR 2.3–5.0)), arthritis did not reoccur, ensuring the sustainability of SDFR.

A limitation is that the biomarker levels were measured with a large interval, due to the structured research visits at which serum was stored. The observed decrease in the levels could have been gradual during 12 months, but may also have occurred within the first weeks or months after DMARD start, concomitantly with the DAS response. A recent study showed that MMP-1 and MMP-3 already declined in the first month after treatment initiation, which might suggest that in the identified subgroup, these biomarkers might also decrease rapidly after DMARD start [16]. Subsequent studies with smaller time intervals after DMARD initiation would be required to determine the time course in more detail.

So far, the biological pathways underlying the disease resolution in RA are unknown [8]. This study identified a subgroup of ACPA-negative RA patients in whom underlying biological pathways leading towards disease resolution might be fundamentally different. MMP-1 and MMP-3 are involved in cartilage degradation and contribute to the infiltration of inflammatory cells, mediated by fibroblast-like synoviocytes (FLS) [17]. FLS are instrumental in creating a microenvironment that favors inflammatory cell retention and the perpetuation of immune pathology in RA [18]. Moreover, multiple studies have shown that the expression of MMP-1 and MMP-3 in FLS can be upregulated by SAA [17, 19]. In turn, SAA, which is believed to be a systemic inflammation marker, can also be locally produced by activated FLS and thus further creating a vicious circle of inflammation [19, 20]. The observed differences in matrix metalloproteases and SAA levels in the first year of therapy might suggest that there are differences at the level of FLS in the identified subgroup within ACPA-negative RA leading towards disease resolution. Molecular studies on tissue level in relation to SDFR could be the next step towards the understanding of the mechanisms related to persistence of ACPA-negative RA.

The markers studied were selected based on previous work, where the biomarkers were mostly selected based on practical argument [9]. Thus, a limitation of this study is that this was not a complete list of possibly relevant markers (for instance, biomarkers representing the T cell and B cell response were underrepresented, and a limited range of interleukins was measured). Therefore, it is presumable that other biomarkers are also appropriate for subgroup identification. Whether other biomarkers could be helpful in further identifying clinically relevant subgroups is a subject for further research.

In our view, this may be the first identification of a clinically relevant subgroup within ACPA-negative RA. Interestingly, and despite the current popularity of big data being explored in a hypothesis-free manner, our findings were done in a hypothesis-driven study. Previous studies that applied hypothesis-free clustering techniques on clinical and MR imaging data at baseline did not identify clear clusters within ACPA-negative RA [6, 7]. Moreover, we demonstrated that ACPA-negative RA patients achieving SDFR can be recognized by high baseline CRP levels and early DAS remission, measures which are both easily accessible in clinical practice. Thus, although the other identified biomarkers (MMP-1/MMP-3/SAA) contributed to the understanding of SDFR in RA, these biomarkers (individually or when combined) are apparently not essential for the clinical identification of these patients. However, the identified subgroup did not include all ACPA-negative RA patients who achieved SDFR. Some ACPA-negative RA patients who achieved SDFR did not have high baseline CRP levels with early DAS remission, and vice versa. This shows the heterogeneity among ACPA-negative RA. Presumably, several biological mechanisms underlie disease resolution, represented by different subgroups within ACPA-negative RA. Hence, our finding may not be sensitive enough to detect all ACPA-negative RA patients who can achieve SDFR.

Interestingly, the identified subgroup had relatively low symptom duration at baseline (Table 2) and achieved early DAS remission. It could therefore be suggested that a short symptom duration is simply associated with early DAS remission and SDFR at the longer course. The fact that this “simple idea” is insufficiently explanatory is shown by the finding of ACPA-negative RA patients with similar short symptom duration and rapid DAS remission, but who did not achieve SDFR (Table 3). This underlines that we have found a specific subgroup, characterized by the combination of early DAS remission and a strong biomarker response. Possibly, not only suppression of clinical disease activity, but also of other inflammatory mechanisms (of which the biomarkers are reflections), lead towards the ability to achieve disease resolution. Importantly, the type of DMARD therapy did not differ between ACPA-negative RA patients achieving early DAS remission and those who did not. Oral steroids as a bridging therapy are of particular interest because of their capacity to induce a strong DAS decline; however, this was similar in both groups (29% vs. 28%).

To search for replication, we studied RA patients consecutively included between 2007 and 2010. The follow-up duration of these patients was sufficient to determine SDFR over time. Tapering and discontinuation of DMARD treatment were less readily done at that time, which was reflected by the finding that SDFR was achieved after a longer treatment duration (median 4.9 years, IQR 3.0–6.8) compared to the study population (median 3.6 years, IQR 2.8–4.9) (Fig. 6). Further validation in RA patients that are treated with current strategies and with sufficient long-term follow-up remains warranted.

It has been suggested that SDFR development in ACPA-negative patients solely reflects the spontaneous resolution of inflammation in patients misclassified as RA [21]. As ACPA-negative RA is treated (and as we did not perform a randomized clinical trial with a placebo arm), we have no data on the natural disease course. However, to prevent that we studied patients with a self-limiting disease, we only analyzed patients who had a clinical RA diagnosis after 1 year of follow-up and additionally fulfilled the 1987 and/or 2010 criteria for RA [12, 13]. Patients diagnosed with other conditions than RA (e.g., reactive arthritis/inflammatory osteoarthritis) were excluded. Thus, according to the current standards, these patients had RA. Second, in sub-analyses, we excluded patients who achieved SDFR within 3 years after DMARD initiation (as this group would presumably include spontaneously remitting patients if these would have been present). This yielded similar results. In our view, this suggests that the early DAS remission, which was identified as a predictor, was related to the DMARD treatment of RA.

## Conclusion

In conclusion, a subgroup of ACPA-negative RA patients with an early DAS response and a strong biomarker response, which achieves SDFR over time, was identified. This subgroup can be clinically recognized by relatively high baseline CRP levels and early DAS remission. Our results might reveal the first clinically relevant subgroup in ACPA-negative RA and indicate a step towards stratified treatment and tapering strategies within ACPA-negative RA.

## Supplementary Information

**Additional file 1: S1.** Flowchart study population and replication population. **S2.** Baseline characteristics of the RA-patients excluded from the study population. **S3.** Statistical methods (extended). **S4.** Baseline characteristics of the RA-patients with and without follow-up biomarker level measurements. **S5.** Baseline characteristics of ACPA-positive RA patients, stratified for SDFR-development. **S6.** ACPA-negative RA-patients achieving SDFR are characterized by a stronger decline in MMP-3, MMP-1, SAA and CRP in the first 12-months after DMARD-initiation. **S7.** In ACPA-positive RA, no differences in course of levels of MMP-3, MMP-1, SAA and CRP were seen between patients achieving SDFR and those who did not. **S8.** Graphs visualizing the relation between baseline levels and delta levels in the other biomarkers. **S9.** The subgroup of ACPA-negative RA achieving SDFR demonstrate a strong clinical and serological response, in contrast to ACPA-negative RA-patients not achieving SDFR or ACPA-positive RA-patients. **S10.** Sub analyses excluding ACPA-negative RA-patients who achieved SDFR <3 years of follow-up showed similar results. **S11.** Sub analyses in ACPA-negative RA-patients initially treated with methotrexate showed similar results. **S12.** Sub analyses in ACPA-negative RA-patients without rheumatoid factor showed similar results.

## Data Availability

All data relevant to the study are included in the article or uploaded as supplementary information. Additional data are available upon reasonable request.

## Abbreviations

ACPA: Anti-citrullinated protein antibodies
CRP: C-reactive protein
DAS: Disease activity score
EGF: Epidermal growth factor
ESR: Estimated sedimentation rate
IL-6: Interleukin-6
MMP: Matrix metalloproteinase
RA: Rheumatoid arthritis
RF: Rheumatoid factor
SAA: Serum amyloid A
SJC: Swollen joint count
SDFR: Sustained DMARD-free remission
TJC: Tender joint count
TNF-R1: Tumor necrosis factor receptor superfamily member 1A
VAS: Visual analog scale
VCAM-1: Vascular cell adhesion molecule-1
VEGF: Vascular endothelial growth factor-A
YKL-40: Human cartilage glycoprotein-39
