# Supplementary material for: Unraveling heterogeneity within ACPA-negative rheumatoid arthritis: the subgroup of patients with a strong clinical and serological response to initiation of DMARD treatment favor disease resolution
Source: Arthritis Res Ther. 2022 Jan 3;24:4. doi: 10.1186/s13075-021-02671-z (PMC8722281; doi:10.1186/s13075-021-02671-z)
Supplement: Supplementary file 1 — Additional file 1: S1. Flowchart study population and replication population. S2. Baseline characteristics of the RA-patients excluded from the study population. S3. Statistical methods (extended). S4. Baseline characteristics of the RA-patients with and without follow-up biomarker level measurements. S5. Baseline characteristics of ACPA-positive RA patients, stratified for SDFR-development. S6. ACPA-negative RA-patients achieving SDFR are characterized by a stronger decline in MMP-3, MMP-1, SAA and CRP in the first 12-months after DMARD-initiation. S7. In ACPA-positive RA, no differences in course of levels of MMP-3, MMP-1, SAA and CRP were seen between patients achieving SDFR and those who did not. S8. Graphs visualizing the relation between baseline levels and delta levels in the other biomarkers. S9. The subgroup of ACPA-negative RA achieving SDFR demonstrate a strong clinical and serological response, in contrast to ACPA-negative RA-patients not achieving SDFR or ACPA-positive RA-patients. S10. Sub analyses excluding ACPA-negative RA-patients who achieved SDFR <3 years of follow-up showed similar results. S11. Sub analyses in ACPA-negative RA-patients initially treated with methotrexate showed similar results. S12. Sub analyses in ACPA-negative RA-patients without rheumatoid factor showed similar results. [file 13075_2021_2671_MOESM1_ESM.pdf]

SUPPLEMENTAL MATERIALS

S1. Flowchart study population and replication population

S2. Baseline characteristics of the RA-patients excluded from the study population

S3. Statistical methods (extended)

S4. Baseline characteristics of the RA-patients with and without follow-up biomarker level measurements

S5. Baseline characteristics of ACPA-positive RA patients, stratified for SDFR-development

S6. ACPA-negative RA-patients achieving SDFR are characterized by a stronger decline in MMP-3, MMP-1, SAA and CRP in the first 12-months after DMARD-initiation

S7. In ACPA-positive RA, no differences in course of levels of MMP-3, MMP-1, SAA and CRP were seen between patients achieving SDFR and those who did not

S8. Graphs visualizing the relation between baseline levels and delta levels in the other biomarkers

S9. The subgroup of ACPA-negative RA achieving SDFR demonstrate a strong clinical and serological response, in contrast to ACPA-negative RA-patients not achieving SDFR or ACPA-positive RA-patients

S10. Sub analyses excluding ACPA-negative RA-patients who achieved SDFR <3 years of follow-up showed similar results

S11. Sub analyses in ACPA-negative RA-patients initially treated with methotrexate showed similar results

S12. Sub analyses in ACPA-negative RA-patients without rheumatoid factor showed similar results

S1 - Flowchart of study population & replication population

A. Selection of study population

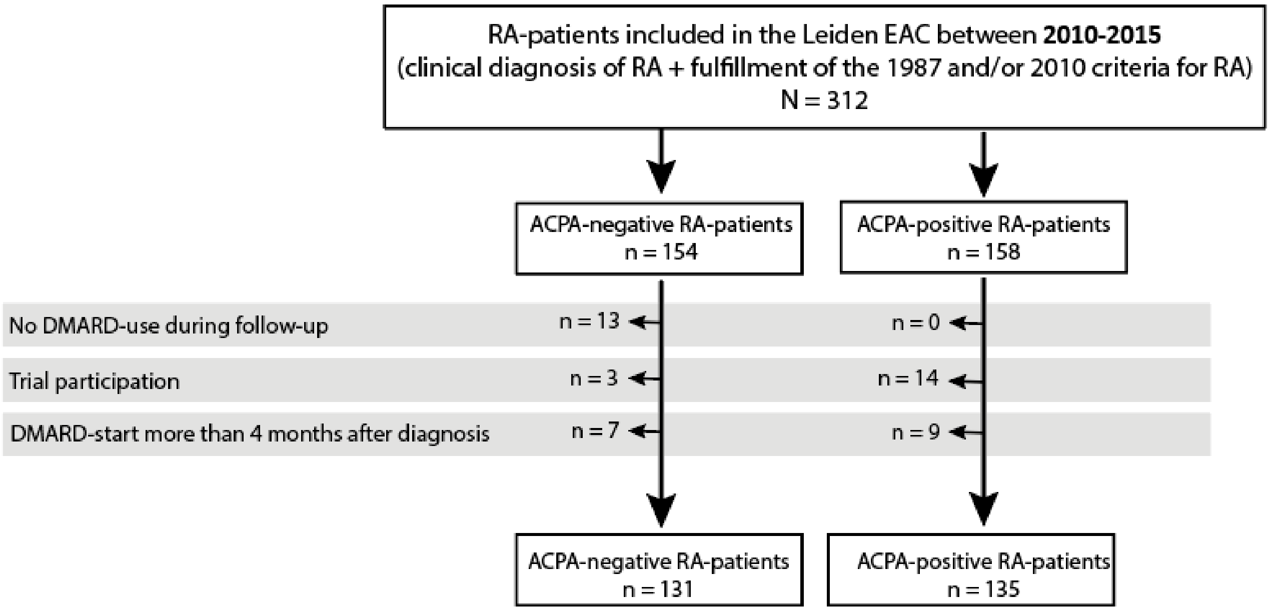

B. Selection of replication population

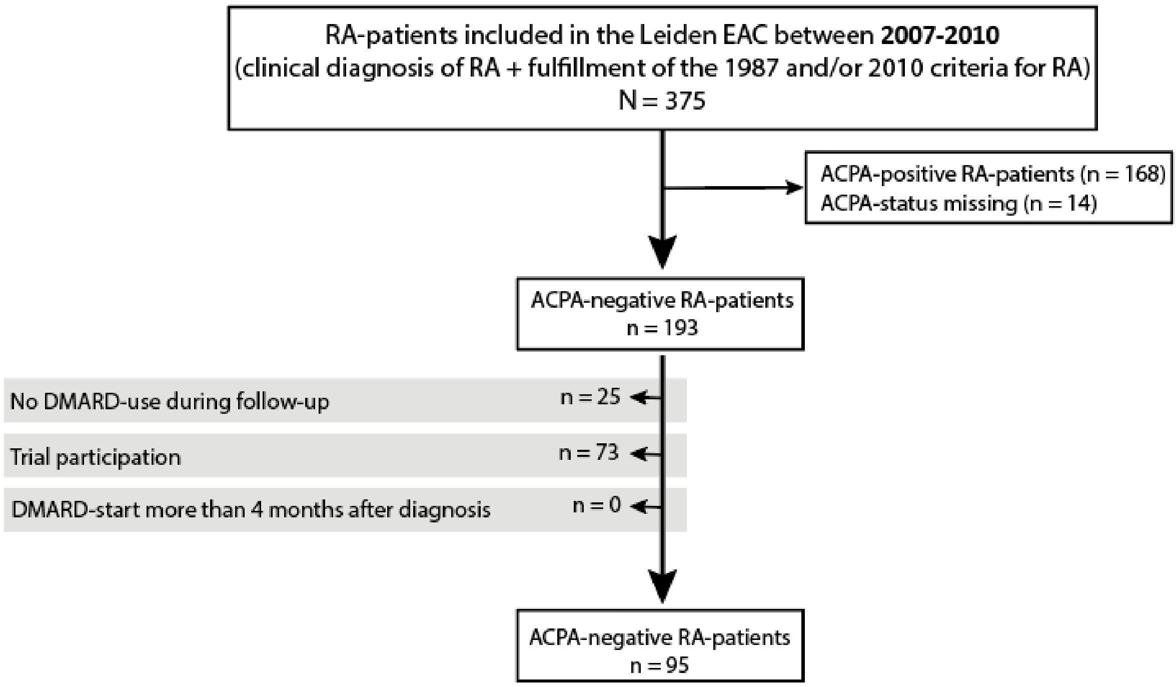

Legend: Flowchart with selection of primary study population (A) and replication population (B). Clinical trials in which RA-patients concomitantly participated included: IMPROVED study, U-act-Early trial and C-early trial. *DMARD: disease modifying antirheumatic drug, EAC: early arthritis clinic, SDFR: sustained DMARD-free remission*

S2 – Baseline characteristics of the RA-patients excluded from the study population

	**Study population** (n=266)	**Excluded RA-patients** (n=46)
Age (years), mean (SD)	57 (15)	54 (13)
Females, %	67	62
ACPA positivity, %	51	50
RF positivity, %	60	54
Symptom duration at diagnosis *(≤12 vs >12w)*, %	40	52
DAS at baseline, med (IQR)	3.4 (2.6 - 4.1)	3.1 (2.5-3.8)
SJC at baseline, (0-44), med (IQR)	5 (2 - 11)	4 (2-9)
TJC at baseline, (0-53), med (IQR)	8 (4 - 15)	9 (4-14)
ESR (mm/h), med (IQR)	28 (14 - 41)	17 (8-35)*
VAS (0 -100 mm), med (IQR)	50 (30 - 70)	50 (20-60)
HAQ, med (IQR)	1.0 (0.5-1.5)	0.9 (0.5-1.4)

Legend: Comparison of baseline characteristics of the study population and the RA-patients excluded from the study population. Despite lower ESR-levels at baseline, baseline characteristics did not significantly differ between both groups. * $p<0.05$

DAS: disease activity score based on swollen joint count (44 joints), tender joint count (53 joints), ESR and pain. SJC: swollen joint count, TJC: tender joint count, ESR: Estimated Sedimentation Rate, VAS: Visual Analogue Scale, RF: Rheumatoid Factor

S3 – Statistical methods (extended)

To test our first hypothesis (figure 1), the course of the twelve individual biomarkers was compared between the SDFR-group and non-SDFR-group using linear mixed models (LMM), stratified for ACPA-status. Splines were used to model the relation between biomarkers levels in the first 2-years after DMARD initiation, with knots at baseline, 12-months and 24-months (based on the timepoints at which biomarker levels were measured). Thus, results reflected change in biomarker levels for two time periods: 0-12 months and 12-24 months. However, whether these changes happened gradually or in a specific part of those time periods could unfortunately not be described since samples were only taken at specific timepoints. Biomarker levels were log-transformed because of non-normal distribution. Results on the logarithmic scale were converted to ratios since these are easier to interpret, namely as decline of biomarker levels in the SDFR-group relative to the decline in levels the non-SDFR group. Because logarithmic results are measured on a multiplicative scale, ratios were retrieved by calculating the difference in decline in biomarker levels in the SDFR-group and non-SDFR-group (on the logarithmic scale) and subsequently exponentiating this difference.

Of the included 266 RA-patients, follow-up biomarker measurements were not available in 42 RA-patients. Thus, solely baseline biomarker information of these RA-patients was included into the models. Missing biomarker measurements were not imputed or excluded since LMM-analysis can handle this missing data, assuming missingness is at random. Baseline characteristics of these 42 RA-patients did not remarkably differ from patients who did have follow-up biomarker data (S4).

As it was previously observed that biomarker levels at diagnosis were related to the chance of achieving SDFR,[1] correlations between baseline biomarker levels and decline in levels in the first 12-months were plotted (hypothesis 2, figure 1), and expressed using Spearman's rho. Additionally, considering previous findings on the relation between early DAS-remission (i.e. DAS<1.6 after 4-months; $DAS_{4months}$) and SDFR,[2] median biomarker change within the first 12-months was compared between RA-patients achieving early DAS-remission, and patients with $DAS_{4months}$ ≥1.6 using Mann-Whitney-U test (hypothesis 3, figure 1). When $DAS_{4months}$ was missing, these were imputed using 8-month DAS-information. Analyses were repeated using non-imputed data to see whether similar results were found (data not shown).

References:

[1] D.M. Boeters et al. ACPA-negative RA consists of subgroups: patients with high likelihood of achieving sustained DMARD-free remission can be identified by serological markers at disease presentation. Arthritis Res Ther 2019;21(1):121.

[2] Verstappen M, Niemantsverdriet E, Matthijssen XME, et al. Early DAS response after DMARD-start increases probability of achieving sustained DMARD-free remission in rheumatoid arthritis. Arthritis Res Ther 2020;22(1):276

S4 – Baseline characteristics of the patients with and without follow-up biomarker measurements

	**Patients with follow-up biomarker measurements** (n=224)	**Patients with no follow-up biomarker measurements** (n=42)
Age (years), mean (SD)	57 (14)	59 (17)
Females, %	66	69
ACPA positivity, %	50	52
RF positivity, %	58	69
Symptom duration at diagnosis *(≤12 vs >12w),* %	41	38
DAS at baseline, med (IQR)	3.3 (2.6 - 4.0)	3.7 (2.7-4.5)
SJC at baseline, (0-44), med (IQR)	5 (2 - 10)	6 (3-14)
TJC at baseline, (0-53), med (IQR)	8 (4 - 15)	11 (4-19)
ESR (mm/h), med (IQR)	28 (14 - 39)	27 (14-51)
VAS (0 -100 mm), med (IQR)	50 (30-70)	60 (30-70)
HAQ, med (IQR)	1.0 (0.5-1.5)	0.9 (0.6-1.3)

Legend: Comparison of baseline characteristics of RA-patients in the study population with follow-up biomarker measurements (n=224) and RA-patients who did not have any follow-up biomarker measurements (n=42). No statistically significant differences in baseline characteristics were seen. * p <0.05

ˣ DAS, ESR, VAS and HAQ at baseline were missing in respectively 7, 1, 6 and 19 patients

DAS: disease activity score based on swollen joint count (44 joints), tender joint count (53 joints), ESR and pain. SJC: swollen joint count, TJC: tender joint count, ESR: Estimated Sedimentation Rate, VAS: Visual Analogue Scale, RF: Rheumatoid Factor

S5 – Baseline characteristics of ACPA-positive RA-patients, stratified for SDFR-development

	**ACPA-positive RA** (n=135)	**No SDFR** (n=126)	**SDFR** (n=9)
Age (years), mean (SD)	64 (11)	54 (14)	59 (10)
Females, %	67	66	78
RF positivity %	84	84	78
Symptom duration at diagnosis *(≤12 vs >12w)*, %	36	37	11
DAS at baseline, med (IQR)	3.2 (2.6 - 3.8)	3.2 (2.6 – 3.9)	3.6 (2.7 - 4.7)
SJC at baseline, (0-44), med (IQR)	5 (2 - 8)	5 (2 - 8)	3 (2 - 7)
TJC at baseline, (0-53), med (IQR)	7 (4 - 12)	7 (4 - 12)	7 (3 - 10)
ESR (mm/h), med (IQR)	29 (14 - 43)	29 (14 - 43)	34 (14 - 39)
VAS (0 -100 mm), med (IQR)	40 (20 - 60)	50 (20-70)	40 (30 - 40)
HAQ, med (IQR)	0.9 (0.4 - 1.4)	0.9 (0.4 - 1.4)	1.1 (0.4 - 1.4)

Legend: Baseline characteristics of ACPA-positive RA-patients achieving SDFR compared to those not achieving SDFR. No significant differences were seen, yet the ACPA-positive group achieving SDFR was relatively small. *p<0.05

DAS: disease activity score based on swollen joint count (44 joints), tender joint count (53 joints), ESR and pain. SJC: swollen joint count, SDFR: Sustained DMARD-free remission, TJC: tender joint count, ESR: Estimated Sedimentation Rate, VAS: Visual Analogue Scale, RF: Rheumatoid Factor

S6 – ACPA-negative RA-patients achieving SDFR are characterized by a stronger decline in MMP-3, MMP-1, SAA and CRP in the first 12-months after DMARD-initiation

MMP-3	**No SDFR** (n=68)	**SDFR** (n=63)	**p-value**	**Exp. difference in log(decline)***	**Ratio of decline in SDFR vs. non-SDFR group****
**Baseline** (log-)MMP-3 (pg/ml)(95% CI)	10.74 (10.54,10.94)	10.80 (10.31,11.29)	0.66	-	-
**Change** in (log-) MMP-3 (95%CI)					
0-12 months	**-0.35 (-0.60, -0.09)**	**-0.71 (-0.96, -0.45)**	**0.048**	**0.70 (0.49-0.97)**	**1.44x (1.00-2.06)**
12-24 months	-0.28 (-0.55, +0.00)	-0.07 (-0.35, +0.22)	0.305	1.23 (0.83-1.83)	0.81x (0.55-1.21)
MMP-1					
**Baseline** (log-)MMP1 (pg/ml) (95%CI)	9.12 (8.95, 9.28)	9.28 (8.88, 9.68)	0.174	-	-
**Change** in (log-) MMP1 (95%CI)					
0-12 months	**-0.04 (-0.17, +0.09)**	**-0.30 (-0.44, -0.17)**	**0.006**	**0.77 (0.64-0.93)**	**1.30x (1.08-1.57)**
12-24 months	-0.06 (-0.20, +0.09)	-0.04 (-0.19, +0.11)	0.836	1.02 (0.83-1.26)	0.98x (0.80-1.20)
Serum A Amyloid					
**Baseline** (log-)SAA (ug/ml) (95% CI)	**1.95 (1.62, 2.29)**	**2.51 (1.70, 3.17)**	**0.023**	-	-
**Change** in (log-)SAA (95%CI)					
0-12 months	**-1.28 (-1.75, -0.81)**	**-2.03 (-2.50, -1.55)**	**0.028**	**0.47 (0.24-0.92)**	**2.12x (1.08-4.14)**
12-24 months	-0.00 (-0.53, +0.52)	+0.07 (-0.47, +0.60)	0.851	1.07 (0.51-2.26)	0.93x (0.44-0.93)
C-reactive protein					
**Baseline** (log-)CRP (ug/ml) (95% CI)	2.68 (2.34, 3.03)	3.00 (2.15, 3.84)	0.216	-	-
**Change** in (log-)CRP (95%CI)					
0-12 months	**-1.46 (-1.93, -1.00)**	**-2.27 (-2.74, -1.80)**	**0.017**	**0.45 (0.23-0.86)**	**2.24x (1.16-4.35)**
12-24 months	-0.04 (-0.56, +0.47)	+0.28 (-0.24, +0.82)	0.379	1.38 (0.73-2.91)	0.72x (0.34-1.50)
IL-6					
**Baseline** (log-)IL-6 (pg/ml) (95% CI)	3.93 (3.66, 4.20)	3.86 (3.20, 4.52)	0.704	-	-
**Change** in (log-)IL-6 (95%CI)					
0-12 months	-1.42 (-1.76, -1.08)	-1.84 (-2.18, -1.49)	0.094	0.66 (0.41-1.07)	1.51x (0.93-2.46)
12-24 months	-0.20 (-0.58, +0.18)	+0.15 (-0.25, +0.54)	0.212	1.41 (0.82-2.44)	0.71x (0.41-1.22)
Resistin					
**Baseline** (log-)Resistin (ng/ml)(95%CI)	2.17 (2.09, 2.26)	2.16 (1.95, 2.36)	0.821	-	-
**Change** in (log-)Resistin (95%CI)					
0-12 months	-0.08 (-0.15, -0.00)	-0.05 (-0.13, +0.02)	0.664	1.02 (0.92-1.14)	0.98x (0.89-1.09)
12-24 months	+0.01 (-0.08, +0.08)	-0.05 (-0.13, +0.04)	0.392	0.95 (0.85-1.07)	1.05x (0.94-1.18)
Leptin					
**Baseline** (log-)Leptin (ng/ml) (95%CI)	2.35 (2.09, 2.61)	2.39 (1.76, 3.02)	0.841	-	-
**Change** in (log-) Leptin (95%CI)					
0-12 months	+0.17 (+0.03, +0.32)	+0.06 (-0.09, +0.20)	0.277	0.89 (0.73-1.10)	1.12x (0.91-1.38)
12-24 months	+0.03 (-0.12, +0.19)	+0.09 (-0.08, +0.25)	0.668	1.05 (0.84-1.32)	0.95x (0.76-1.20)

YKL-40	**No SDFR** (n=68)	**SDFR** (n=63)	**p-value**	**No SDFR** (n=68)	**SDFR** (n=63)
**Baseline** (log-)YKL-40 (pg/ml)(95%CI)	11.47 (11.30,11.63)	11.51 (11.1,11.91)	0.735	-	-
**Change** in (log-) YKL-40 (95%CI)					
0-12 months	-0.35 (-0.52, -0.18)	-0.18 (-0.35, -0.01)	0.177	1.18 (0.93-1.50)	0.85x (0.67-1.08)
12-24 months	+0.08 (-0.11, +0.26)	+0.01 (-0.19, +0.20)	0.615	0.93 (0.71-1.22)	1.07x (0.82-1.40)
TNF-R1					
**Baseline** (log-)TNF-R1 (ng/ml) (95%CI)	0.53 (0.45, 0.61)	0.56 (0.36, 0.77)	0.608	-	-
**Change** in (log-) TNF-R1 (95%CI)					
0-12 months	-0.12 (-0.21, -0.03)	-0.15 (-0.23, +0.06)	0.667	0.97 (0.86-1.10)	1.03x (0.91-1.16)
12-24 months	-0.00 (-0.09, +0.10)	-0.00 (-0.10, +0.10)	0.964	1.00 (0.87-1.15)	1.00x (0.87-1.14)
EGF					
**Baseline** (log-)EGF (pg/ml) (95% CI)	5.29 (5.13, 5.44)	5.22 (4.85, 5.58)	0.540	-	-
**Change** in (log-)EGF (95%CI)					
0-12 months	+0.01 (-0.17, +0.19)	-0.02 (-0.20, +0.16)	0.818	0.97 (0.76-1.25)	1.03x (0.80-1.32)
12-24 months	-0.15 (-0.34, +0.05)	-0.01 (-0.21, +0.20)	0.323	1.15 (0.87-1.53)	0.87x (1.15-0.66)
VEGF					
**Baseline** (log-)VEGF (pg/ml) (95% CI)	5.91 (5.76, 6.06)	5.93 (5.57, 6.30)	0.861	-	-
**Change** in (log-)VEGF (95%CI)					
0-12 months	-0.33 (-0.43, -0.23)	-0.37 (-0.47, -0.28)	0.545	0.96 (0.83-1.10)	1.04x (0.91-1.20)
12-24 months	-0.04 (-0.15, +0.07)	-0.04 (-0.15, +0.08)	0.948	1.01 (0.86-1.17)	1.00x (0.85-1.16)
VCAM-1					
**Baseline** (log-)VCAM-1 (pg/ml)(95%CI)	13.52 (13.46,13.58)	13.49 (13.34,13.65)	0.555	-	-
**Change** in (log-) VCAM-1 (95%CI)					
0-12 months	-0.15 (-0.22, -0.09)	-0.13 (-0.19, -0.07)	0.636	1.02 (0.93-1.12)	0.98x (0.90-1.07)
12-24 months	+0.04 (-0.03, +0.11)	+0.04 (-0.03, +0.11)	0.940	1.00 (0.90-1.10)	1.00x (0.91-1.11)

Legend: Estimated marginals means for (log-)decline in biomarker levels between baseline and 12-months and between 12-24 resulting from linear mixed model analyses of all twelve biomarkers over time in relation to SDFR-development in ACPA-negative RA.
* Decline in biomarker levels (on the logarithmic scale) in the SDFR-group subtracted by the decline in biomarker levels in the non-SDFR group; e.g Δ0-12m(log)SAA_{SDFR} – Δ0-12m(log)$SAA_{non\text{-}SDFR}$, and subsequently exponentiated.
** Ratio of decline of biomarker levels in the SDFR-group relative to the decline of biomarker levels in the non-SDFR group, calculated by 1/(exponentiated difference in (log)decline between both groups).

CRP: C-reactive protein, SAA: serum amyloid A, TNF-R1: tumor necrosis factor receptor superfamily member 1A (TNFR1), IL-6: interleukin-6, MMP: matrix metalloproteinase, EGF: epidermal growth factor, VEGF: vascular endothelial growth factor-A, VCAM-1: vascular cell adhesion molecule-1, YKL-40: human cartilage glycoprotein-39

S7 – In ACPA-positive RA, no differences in course of levels of MMP-3, MMP-1, SAA and CRP were seen between patients achieving SDFR and those who did not

Serum A Amyloid	**No SDFR** (n=126)	**SDFR** (n=9)	**p-value**	**Exp. difference in log(decline)***	**Ratio of decline in SDFR vs. non-SDFR group****
**Baseline** (log-)SAA (ug/ml) (95% CI)	1.67 (1.43, 1.95)	1.71 (0.55, 2.91)	0.934	-	-
**Change** in (log-)SAA (95%CI)					
0-12 months	-1.06 (-1.37, -1.37)	-1.19 (-2.27, +0.11)	0.816	0.88 (0.28-2.70)	1.14x (0.37-3.52)
12-24 months	+0.05 (-0.28, +0.38)	+0.56 (-0.51, +1.63)	0.377	1.67 (0.54-5.10)	0.60x (0.20-1.85)
C-reactive protein					
**Baseline** (log-)CRP (ug/ml) (95% CI)	2.46 (2.21, 2.70)	1.86 (0.67, 3.04)	0.213	-	-
**Change** in (log-)CRP (95%CI)					
0-12 months	-1.25 (-1.59, -0.91)	-0.64 (-1.80, +0.52)	0.323	1.84 (0.55-6.15)	0.54x (0.16-1.82)
12-24 months	+0.15 (-0.20, +0.51)	+0.39 (-0.76, +1.54)	0.697	1.27 (0.38-4.24)	0.79x (0.24-2.62)
MMP-1					
**Baseline** (log-)MMP1 (pg/ml) (95%CI)	9.05 (8.92, 9.17)	9.00 (8.39, 9.61)	0.861	-	-
**Change** in (log-) MMP1 (95%CI)					
0-12 months	-0.04 (-0.12, +0.04)	+0.20 (-0.07, +0.47)	0.087	1.28 (0.96-1.70)	0.78x (0.59-1.04)
12-24 months	-0.06 (-0.14, +0.03)	-0.27 (-0.54, -0.01)	0.134	0.81 (0.61-1.07)	1.24x (0.94-1.65)
MMP-3					
**Baseline** (log-)MMP-3 (pg/ml)(95% CI)	10.43 (10.30,10.55)	10.39 (9.78,10.99)	0.874	-	-
**Change** in (log-) MMP-3 (95%CI)					
0-12 months	-0.26 (-0.42, -0.10)	-0.48 (-1.02, +0.06)	0.444	0.80 (0.46-1.41)	1.25x (0.71-2.19)
12-24 months	+0.00 (-0.17, +0.27)	-0.04 (-0.57, +0.50)	0.895	0.96 (0.55-1.69)	1.04x (0.59-1.82)

Legend: Estimated marginals means for decline in biomarker levels between baseline and 12-months and between 12-24 resulting from linear mixed model analyses of all twelve biomarkers over time in relation to SDFR-development in ACPA-positive RA.
* Decline in biomarker levels (on the logarithmic scale) in the SDFR-group subtracted by the decline in biomarker levels in the non-SDFR group; e.g Δ0-12m(log)SAA_{SDFR} – Δ0-12m(log)$SAA_{non\text{-}SDFR}$, and subsequently exponentiated.
** Ratio of decline of biomarker levels in the SDFR-group relative to the decline of biomarker levels in the non-SDFR group, calculated by 1/(exponentiated difference in (log)decline between both groups).

CRP: C-reactive protein, SAA: serum amyloid A, TNF-R1: tumor necrosis factor receptor superfamily member 1A (TNFR1), IL-6: interleukin-6, MMP: matrix metalloproteinase, EGF: epidermal growth factor, VEGF: vascular endothelial growth factor-A, VCAM-1: vascular cell adhesion molecule-1, YKL-40: human cartilage glycoprotein-39

S8. Graphs visualising the relation between baseline levels and delta levels in the other biomarkers

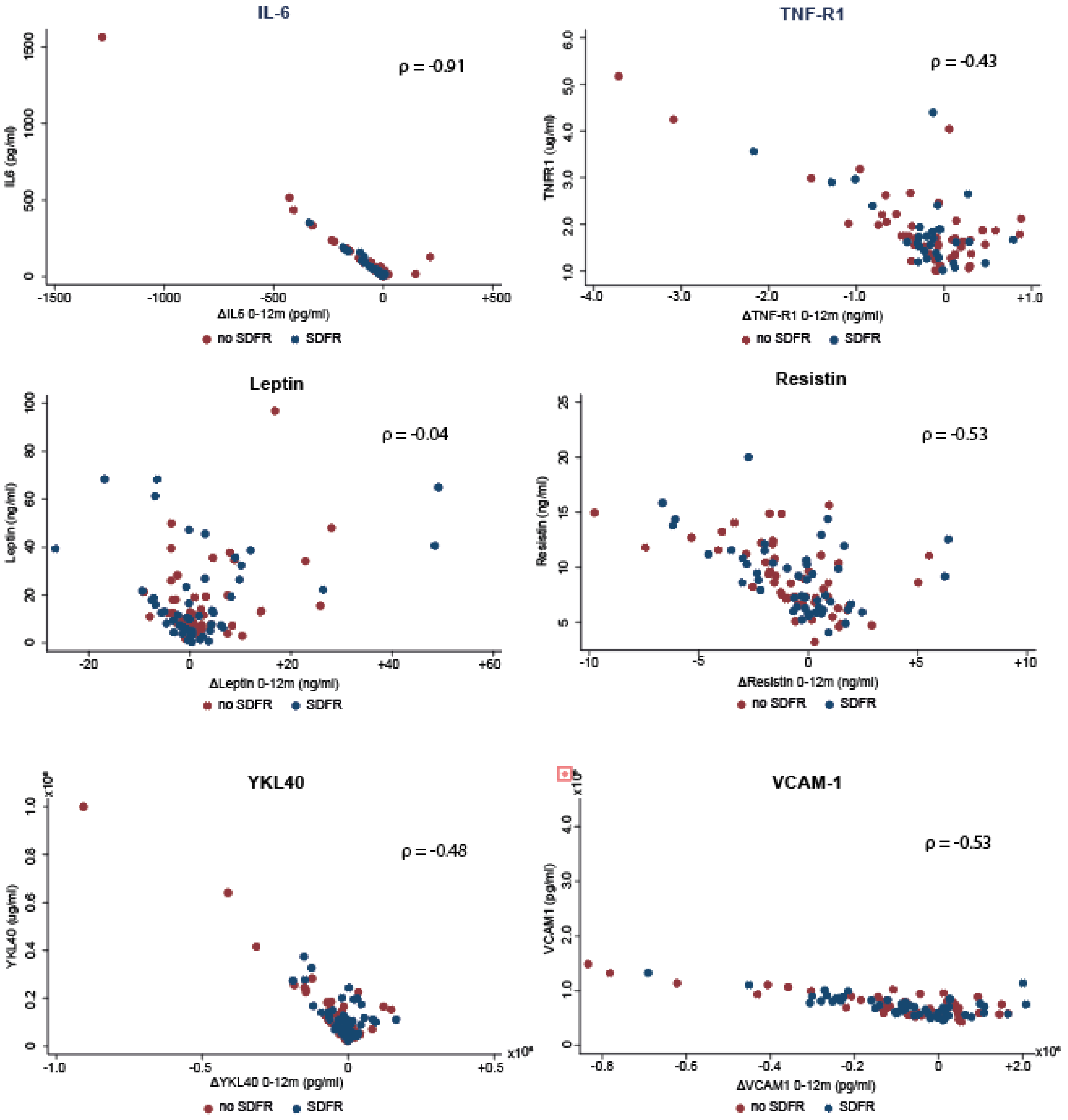

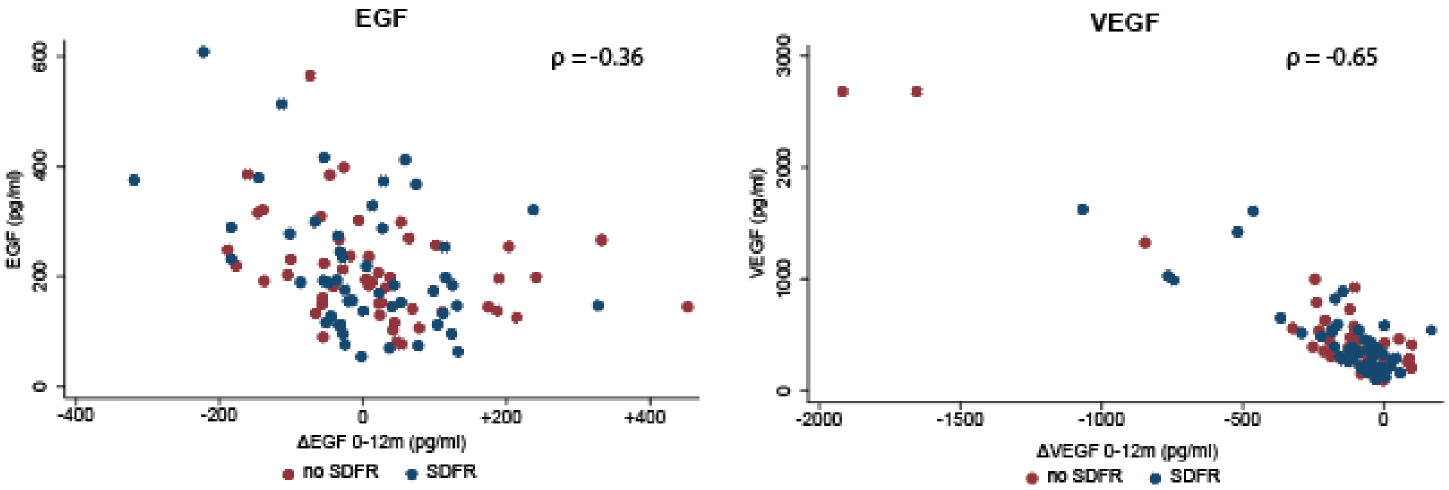

Legend: Graphs of the relation between baseline biomarker levels and change in levels in the first year after treatment initiation, separate for ACPA-negative RA-patients achieving SDFR (n=68) and those who did not (n=63).

ρ reflects Spearmen's rho.

TNF-R1: tumor necrosis factor receptor superfamily member 1A (TNFR1), IL-6: interleukin-6, EGF: epidermal growth factor, VEGF: vascular endothelial growth factor-A, VCAM-1: vascular cell adhesion molecule-1, YKL-40: human cartilage glycoprotein-39, SDFR: sustained DMARD-free remission.

S9. The median biomarker-decline in the first year of therapy is stronger in ACPA-negative RA-patients who achieve early DAS-remission, especially in those achieving SDFR.

A. Median change in biomarker-levels in the first year in relation to early DAS-remission in ACPA-negative RA

	$DAS_{4months}$≥1.6 (n=57)	$DAS_{4months}$<1.6 (n=38)	p-value
**MMP-1 change 0-12m** (pg/ml), *median (IQR)*	-0.1 (-3.6, +1.7) x10^3	-2.2 (-5.0, -0.1) x10^3	0.024
**MMP-3 change 0-12m** (pg/ml), *median (IQR)*	-5.0 (-29.2, +2.5) x10^3	-12.9 (-70.8, +1.8) x10^3	0.186
**SAA change 0-12m** (ug/ml), *median (IQR)*	-3.2 (-18.7, -0.1)	-11.3 (-52.5, -1.9)	0.014
**CRP change 0-12m** (ug/ml), *median (IQR)*	-5.1 (-43.1, +0.0)	-18.0 (-53.3, -5.6)	0.028

B. Median change in biomarker-levels in the first year in relation to early DAS-remission in ACPA-negative RA, stratified for SDFR

ACPA-negative RA	No SDFR			SDFR		
	$DAS_{4months}$≥1.6 (n=33)	$DAS_{4months}$<1.6 (n=16)	p-value	$DAS_{4months}$≥1.6 (n=24)	$DAS_{4months}$<1.6 (n=22)	p-value
**MMP-1 change 0-12m** (pg/ml), *median (IQR)*	+0.7 (-3.3,+2.6) x10^3	-0.6 (-3.4,+0.7) x10^3	0.171	-0.6 (-4.9, +0.4) x10^3	**-2.9 (-6.4,-1.3) x10^3**	0.077
**MMP-3 change 0-12m** (pg/ml), *median (IQR)*	-8.1 (-30.0,+2.2) x10^3	-5.4 (-25.6 +5.0) x10^3	0.787	-4.4 (-34.8,-31.6) x10^3	**-31.6 (-82.3,-9.0) x10^3**	0.027
**SAA change 0-12m** (ug/ml), *median (IQR)*	-3.0 (-24.2, -0.0)	-4.0 (-41.0, -1.6)	0.223	-3.6 (-21.3, -0.0)	**-18.1 (-55.8, -7.1)**	0.051
**CRP change 0-12m** (ug/ml), *median (IQR)*	-4.3 (-43.1, +1.0)	-9.5 (-58.2, -3.0)	0.113	-8.9 (-43.5, -0.9)	**-28.0 (-53.3, -11.7)**	0.151

C. Median change in biomarker-levels in the first year in relation to early DAS-remission in ACPA-positive RA

	No SDFR			SDFR		
	$DAS_{4months}$≥1.6 (n=60)	$DAS_{4months}$<1.6 (n=23)	p-value	$DAS_{4months}$≥1.6 (n=5)	$DAS_{4months}$<1.6 (n=4)	p-value
**MMP-1 change 0-12m** (pg/ml), *median (IQR)*	-0.3 (-2.0, +1.5) x10^3	+0.1 (-17.7 +4.3) x10^3	0.610	+1.8 (-0.0, +4.8) x10^3	+1.6 (-0.1, +2.9) x10^3	0.905
**MMP-3 change 0-12m** (pg/ml), *median (IQR)*	+1.0 (-19.4,+8.4) x10^3	-4.2 (-38.5, +2.6) x10^3	0.119	-6.9 (-23.1, -0.2) x10^3	+2.0 (31.1, +5.0) x10^3	0.556
**SAA change 0-12m** (ug/ml), *median (IQR)*	-2.0 (-9.9, -0.0)	-1.1 (-3.9, -0.4)	0.568	-10.3 (-16.8, +0.3)	-0.1 (-49.9, +0.1)	0.905
**CRP change 0-12m** (ug/ml), *median (IQR)*	-5.5 (-24.6, -0.1)	-5.3 (-16.7, -0.7)	0.758	-4.5 (-44.1, +0.7)	+0.2 (-9.1, +0.3)	0.905

Legend: Median decline in levels of MMP-1, MMP-3, SAA and CRP within the first year after diagnosis is significantly stronger in ACPA-negative RA-patients with early DAS-remission (A), especially in ACPA-negative RA-patients achieving SDFR (B). The combination of a strong clinical (early DAS-remission) and serological response demarcates the subgroup within ACPA-negative RA confined to achieve disease resolution. Such effects are absent in ACPA-positive RA (C). *CRP: C-reactive protein, MMP: matrix metalloproteinase, SAA: serum A amyloid, SDFR: sustained DMARD-free remission.*

S10. Sub analyses excluding ACPA-negative RA-patients who achieved SDFR <3 years of follow-up showed similar results

A. Linear mixed model results of differences in course of MMP-1, MMP-3, SAA and CRP between the SDFR and non-SDFR group.

Serum A Amyloid	**No SDFR** (n=68)	**SDFR** (n=40)	**p-value**	**Exp. difference in log(decline)***	**Ratio of decline in SDFR vs. non-SDFR group****
**Baseline** (log-)SAA (ug/ml) (95% CI)	1.95 (1.61, 2.30)	2.28 (1.36, 3.20)	0.262	-	-
**Change** in (log-)SAA (95%CI)					
0-12 months	-1.28 (-1.76, -0.80)	-1.81 (-2.44, -1.19)	0.184	0.59 (0.27-1.29)	1.71x (0.78-3.75)
12-24 months	-0.00 (-0.53, +0.53)	+0.02 (-0.62, +0.67)	0.951	1.03 (0.45-2.36)	0.97x (2.24-0.42)
C-reactive protein					
**Baseline** (log-)CRP (ug/ml) (95% CI)	2.68 (2.33, 2.04)	2.84 (1.90, 3.78)	0.597	-	-
**Change** in (log-)CRP (95%CI)					
0-12 months	-1.46 (1.94, -0.98)	-2.11 (-2.73, -1.48)	0.107	0.52 (0.24-1.15)	1.91x (0.87-4.18)
12-24 months	-0.04 (-0.57, +0.49)	+0.26 (-0.38, +0.90)	0.472	1.35 (0.59-3.11)	0.73x (0.32-1.69)
MMP-1					
**Baseline** (log-)MMP1 (pg/ml) (95%CI)	9.12 (8.95, 9.28)	9.32 (8.88, 9.75)	0.144	-	-
**Change** in (log-) MMP1 (95%CI)					
0-12 months	-0.04 (-0.18, +0.09)	-0.22 (-0.39, -0.05)	0.106	0.83 (0.67-1.04)	1.20x (0.96-1.49)
12-24 months	-0.06 (-0.21, +0.09)	-0.08 (-0.26, +0.10)	0.857	0.98 (0.78-1.23)	1.02x (0.81-1.29)
MMP-3					
**Baseline** (log-)MMP-3 (pg/ml)(95% CI)	10.74 (10.53,10.94)	10.73 (10.19,11.26)	0.944	-	-
**Change** in (log-) MMP-3 (95%CI)					
0-12 months	-0.35 (-0.60, -0.09)	-0.66 (-0.99, -0.32)	0.149	0.73 (0.48-1.12)	1.37x (0.89-2.08)
12-24 months	-0.28 (-0.56, +0.01)	-0.16 (-0.51, +0.18)	0.619	1.12 (0.72-1.74)	0.89x (0.57-1.39)

Legend: Estimated marginals means for decline in biomarker levels between baseline and 12-months and between 12-24 resulting from linear mixed model analyses of all twelve biomarkers over time in relation to SDFR-development with exclusion of patients who achieved SDFR within 3 years of follow-up (n=23).
** Decline in biomarker levels (on the logarithmic scale) in the SDFR-group subtracted by the decline in biomarker levels in the non-SDFR group; e.g Δ0-12m(log)SAA$_{SDFR}$ – Δ0-12m(log)SAA$_{non\text{-}SDFR}$, and subsequently exponentiated. ** Ratio of decline of biomarker levels in the SDFR-group relative to the decline of biomarker levels in the non-SDFR group, calculated by 1/(exponentiated difference in (log)decline between both groups).*

B. Relation between baseline levels and decline in baseline and 12-months

	**Spearman's rho**
**SAA**	-0.89
**CRP**	-0.89
**MMP-1**	-0.49
**MMP-3**	-0.62

Legend: Relation between baseline biomarker levels and change in levels in the first year after treatment initiation in patients initially treated with methotrexate.
CRP: C-reactive protein, SAA: serum amyloid A, MMP: matrix metalloproteinase

Abbreviations:
CRP: C-reactive protein, SAA: serum amyloid A, MMP: matrix metalloproteinase

S11. Sub analyses in ACPA-negative RA-patients initially treated with methotrexate

A. Linear mixed model results of differences in course of MMP-1, MMP-3, SAA and CRP between the SDFR and non-SDFR group.

Serum A Amyloid	**No SDFR** (n=55)	**SDFR** (n=44)	**p-value**	**Exp. difference in log(decline)***	**Ratio of decline in SDFR vs. non-SDFR group****
**Baseline** (log-)SAA (ug/ml) (95% CI)	2.00 (1.62, 2.37)	2.47 (1.54, 3.41)	0.097	-	-
**Change** in (log-)SAA (95%CI)					
0-12 months	-1.22 (-1.75, -0.68)	**-2.07 (-2.67, -1.48)**	**0.036**	**0.42 (0.19-0.95)**	**2.36x (1.06-5.26)**
12-24 months	-0.04 (-0.63, +0.55)	+0.11 (-0.54, +0.76)	0.741	1.16 (0.48-2.79)	0.86x (0.36-2.08)
C-reactive protein					
**Baseline** (log-)CRP (ug/ml) (95% CI)	2.75 (2.36-3.14)	3.00 (2.03-3.97)	0.404	-	-
**Change** in (log-)CRP (95%CI)					
0-12 months	-1.40 (-1.92, -0.89)	**-2.31 (-2.88, -1.75)**	**0.020**	**0.40 (0.19-0.87)**	**2.49x (1.15-5.36)**
12-24 months	-0.12 (-0.69, +0.44)	+0.32 (-0.24, +0.10)	0.298	1.56 (0.67-3.62)	0.64x (0.28-1.48)
MMP-1					
**Baseline** (log-)MMP1 (pg/ml) (95%CI)	9.21 (9.03, 9.38)	9.30 (8.87, 9.74)	0.465	-	-
**Change** in (log-) MMP1 (95%CI)					
0-12 months	-0.03 (-0.16, +0.12)	-0.21 (-0.36, +0.05)	0.089	0.83 (0.67-1.03)	1.21x (0.97-1.49)
12-24 months	-0.07 (-0.23, +0.09)	-0.07 (-0.46, +0.32)	0.989	1.00 (0.79-1.26)	1.00x (0.79-1.26)
MMP-3					
**Baseline** (log-)MMP-3 (pg/ml)(95% CI)	10.70 (10.48,10.92)	10.84 (10.28,11.40)	0.408	-	-
**Change** in (log-) MMP-3 (95%CI)					
0-12 months	-0.26 (-0.53, +0.02)	**-0.76 (-1.07, -0.45)**	**0.016**	**0.60 (0.40-0.91)**	**1.66x (1.10-2.50)**
12-24 months	-0.25 (-0.56, +0.05)	-0.03 (-0.36, +0.30)	0.335	1.25 (0.80-1.95)	0.80x (0.51-1.25)

Legend: Estimated marginals means for decline in biomarker levels between baseline and 12-months and between 12-24 resulting from linear mixed model analyses of all twelve biomarkers over time in relation to SDFR-development in ACPA-negative RA-patients initially treated with methotrexate (n=99)

** Decline in biomarker levels (on the logarithmic scale) in the SDFR-group subtracted by the decline in biomarker levels in the non-SDFR group; e.g Δ0-12m(log)SAA$_{SDFR}$ – Δ0-12m(log)SAA$_{non\text{-}SDFR}$, and subsequently exponentiated.*

*** Ratio of decline of biomarker levels in the SDFR-group relative to the decline of biomarker levels in the non-SDFR group, calculated by 1/(exponentiated difference in (log)decline between both groups).*

B. Relation between baseline levels and decline in baseline and 12-months

	Spearman's rho
**SAA**	-0.91
**CRP**	-0.90
**MMP-1**	-0.52
**MMP-3**	-0.66

Legend: Relation between baseline biomarker levels and change in levels in the first year after treatment initiation in patients initially treated with methotrexate.

Abbreviations:

CRP: C-reactive protein, SAA: serum amyloid A, MMP: matrix metalloproteinase

S12. Sub analyses in ACPA-negative RF-negative RA-patients showed similar results

A. Linear mixed model results of differences in course of MMP-1, MMP-3, SAA and CRP between the SDFR and non-SDFR group.

Serum A Amyloid	**No SDFR** (n=35)	**SDFR** (n=49)	**p-value**	**Exp. difference in log(decline)***	**Ratio of decline in SDFR vs. non-SDFR group****
**Baseline** (log-)SAA (ug/ml) (95% CI)	1.80 (1.36, 2.24)	**2.65 (1.63, 3.68)**	**0.004**	-	-
**Change** in (log-)SAA (95%CI)					
0-12 months	-1.14 (-1.79, -0.48)	**-2.17 (-2.71, -1.62)**	**0.018**	**0.36 (0.15-0.84)**	**2.80x (1.19-6.58)**
12-24 months	-0.00 (-0.79, +0.65)	+0.00 (-0.60, +0.60)	0.894	1.07 (0.42-2.73)	0.94x (0.37-2.40)
C-reactive protein					
**Baseline** (log-)CRP (ug/ml) (95% CI)	2.62 (2.13, 3.08)	3.08 (2.13, 4.20)	0.072	-	-
**Change** in (log-)CRP (95%CI)					
0-12 months	-1.26 (-1.91, -0.60)	**-2.42 (-2.96, -1.88)**	**0.007**	**0.31 (0.13-0.73)**	**3.20x (1.37-7.47)**
12-24 months	-0.16 (-0.88, +0.56)	+0.22 (-0.41, +0.82)	0.424	1.46 (0.57-3.73)	0.68x (0.27-1.74)
MMP-1					
**Baseline** (log-)MMP1 (pg/ml) (95%CI)	9.09 (8.87, 9.31)	9.36 (8.85, 9.88)	0.064	-	-
**Change** in (log-) MMP1 (95%CI)					
0-12 months	-0.04 (-0.23, +0.15)	**-0.36 (-0.52, +0.20)**	**0.011**	**0.73 (0.57-0.93)**	**1.38x (1.08-1.76)**
12-24 months	-0.06 (-0.27, +0.14)	-0.04 (-0.19, +0.16)	0.739	1.05 (0.80-1.37)	0.95x (0.73-1.25)
MMP-3					
**Baseline** (log-)MMP-3 (pg/ml)(95% CI)	10.85 (10.55,11.14)	10.85 (10.18,11.53)	0.961	-	-
**Change** in (log-) MMP-3 (95%CI)					
0-12 months	-0.49 (-0.82, -0.15)	-0.72 (-1.00, -0.44)	0.292	0.79 (0.51-1.22)	1.26x (0.82-1.96)
12-24 months	-0.31 (-0.68, +0.06)	-0.13 (-0.44, +0.18)	0.475	1.19 (0.74-1.93)	0.84x (0.52-1.36)

Legend: Estimated marginals means for decline in biomarker levels between baseline and 12-months and between 12-24 resulting from linear mixed model analyses of all twelve biomarkers over time in relation to SDFR-development in ACPA-negative RA-patients initially treated with methotrexate (n=99)

** Decline in biomarker levels (on the logarithmic scale) in the SDFR-group subtracted by the decline in biomarker levels in the non-SDFR group; e.g $\Delta 0\text{-}12m(log)SAA_{SDFR} - \Delta 0\text{-}12m(log)SAA_{non\text{-}SDFR}$, and subsequently exponentiated.*

*** Ratio of decline of biomarker levels in the SDFR-group relative to the decline of biomarker levels in the non-SDFR group, calculated by 1/(exponentiated difference in (log)decline between both groups).*

B. Relation between baseline levels and decline in baseline and 12-months

	**Spearman's rho**
**SAA**	-0.95
**CRP**	-0.96
**MMP-1**	-0.57
**MMP-3**	-0.70

Legend: Relation between baseline biomarker levels and change in levels in the first year after treatment initiation in patients initially treated with methotrexate.

Abbreviations:

CRP: C-reactive protein, SAA: serum amyloid A, MMP: matrix metalloproteinase
